# Self-Perceived Competence of Ambulance Nurses in the Care of Patients with Mental Illness: A Questionnaire Survey

**DOI:** 10.3390/nursrep12010023

**Published:** 2022-03-18

**Authors:** Sandra Önnheim, Anders Johansson, Bodil Ivarsson, Caroline Hagström

**Affiliations:** 1 Office of Medical Services, Region Skane, Kioskgatan 17, 22185 Lund, Sweden; sandra.onnheim@skane.se (S.Ö.); anders.johansson@med.lu.se (A.J.); caroline.hagstrom@skane.se (C.H.); 2 Department of Clinical Sciences, Lund University, P.O. Box 117, 22185 Lund, Sweden

**Keywords:** ambulance services, competence, assessment experience, knowledge, prehospital emergency care, mental illness, nurse, prehospital emergency care, skills

## Abstract

Ambulance nurses in prehospital emergency care must assess, treat, and triage patients with mental health issues. This study aimed to investigate the self-perceived competence of ambulance nurses in prehospital emergency care of patients with mental illness. A cross-sectional questionnaire survey was done, a question-index value (Q-IV; range: 0–1.0) was defined as a summary of the proportion of positive responses (%). Correlations of self-perceived competence with education and professional experience were also examined. Overall self-perceived competence was good (mean Q-IV, 0.80). For six of the nine questions, women rated their abilities slightly lower than men. Women rated themselves as fairly good in providing “information about types of effective help available” (Q-IV, 0.55) and in “suggesting tactics for helping a person with mental illness feel better” (Q-IV, 0.56). Men rated their competence as fairly good in “directing patients to appropriate sources of help” (Q-IV, 0.58). Self-perceived competence did not correlate with education level or professional experience. In conclusion, these results indicate that in encounters with patients who have mental illness, ambulance nurses perceive their overall competencies as good, with some sex-based differences in self-perception for specific knowledge areas. Education level and professional experience did not correlate with self-perceived competence.

## 1. Introduction

Ambulance care is a branch of prehospital care, i.e., provided before the patient arrives at the hospital, administered at the pick-up point, or in an ambulance during transport [1]. In Sweden, ambulance services are staffed with at least one registered nurse (RN), who completed 3 years of undergraduate education to earn a bachelor’s of science in nursing [2]. In Sweden, some regions’ ambulances are also staffed with nurses who have a residency education at an advanced level, including a one-year master’s of science degree. These nurses are designated as specialist nurses (SNs) [3].

Working as a nurse in ambulance care is challenging because the prehospital environment is unpredictable, and the nurse is often the sole decision-maker regarding measures to take for the patient [3,4]. For each patient, the nurse must perform a series of assessments while also collecting background information, evaluating the information, and taking it together with the clinical findings to make a determination regarding immediate care and treatment [1]. In addition, the nurse must assess whether the patient requires continued care and how transport to a department should be performed [5]. Different terms in mental health and/or and alcohol and other drug problems spectra are often used, and in this study the umbrella concept of mental illness will be used.

A Danish study showed that emergency calls to emergency dispatch centers because of mental health crises were extensive, accounting for more than one-third of all incoming calls, and a lot of patients with mental illness are both frequent callers and recurrent users of ambulance service [6]. When a patient presents with potential mental health issues, the situation is further complicated. The symptoms can manifest in several ways, and the ambulance nurse must interpret and evaluate the patient’s expression of them [7]. This variability often is accompanied by a lack of background information because the ambulance service does not have access to previous medical records. These features make assessment, treatment, and planning difficult, even more so as the nurse often does it all solo [4]. When patients have symptoms of mental illness in the prehospital environment, other aggravating components often are present, including need for immediate care for a patient who cannot understand the need or resists it. For this reason, the nurse also must have knowledge and awareness about mandatory measures and legal support in these situations [8]. In addition, the physical work environment is often unsafe, and nurses must engage in an ongoing assessment of their own safety risks [4]. Perrone McIntosh reported that nurses often have an exaggerated belief that patients with mental illness are aggressive, leading the nurses to avoid responding to or asking the questions needed to provide good and equitable care [9]. Petzäll et al. showed that 66% of ambulance personnel had been threatened by a patient or close relative in the past year, most commonly by people under the influence of alcohol and drugs, often on weekends, and by a man in 90% of cases [10].

Mental illness affects many in the population, and every participant in the entire care chain need to be well informed about how to best help and care for patients with these problems, regardless of how severe the mental illness is judged to be. The regular contact routes must be evaluated and are in the process of development so that these patients can receive adequate ambulance care in acute psychiatric stages and sometimes also with the help of destined psychiatric ambulances [7,11,12]. However, generally for ambulance staff, mental illnesses are difficult to manage because of the wide variety within the mental health spectra, lack of collective information and knowledge, and lack of support and cooperation with other care agencies.

Professional competence has been described as the ability to use knowledge and skills in a certain professional context. The transfer of theoretical knowledge and skills into useful competences is often influenced by the professional environment and personal abilities [13]. However, professional competence can also include professional attitudes, approaches, and perceptions found within the profession itself [14]. These different parts of a professional competence can be considered as central when understanding a professional competence in a specific setting [15]. Acute assignments involving mental illness thus require ambulance staff to have well-developed competencies and skills [4]. In this study, we aimed to describe how nurses perceive their own competence regarding the care of patients with mental illness in the prehospital emergency care setting and to describe some relevant characteristics.

## 2. Methods

In this prospective cross-sectional study, we used a questionnaire survey with fixed response options. The study followed the Strengthening the Reporting of Observational Studies in Epidemiology (STROBE) checklist for cross-sectional studies (see Supplementary Materials File S1).

### 2.1. Participants and Data Collection

A survey was distributed to 311 nurses, representing the total population of RNs and SNs in the northwest, northeast, and central Skåne ambulance districts in southern Sweden. Participants received the questionnaires (Appendix A)—including a letter with information about the study, estimated time required, and that it was voluntary to participate—in connection with the start or end of work shifts. By completing the questionnaire and returning it to the contact person in each ambulance district, the participants approved their participation in the study. The contact persons were responsible for collecting the completed questionnaires and returning them to the authors. The data collection took place between January 2021 and March 2021. Reminders were sent once to the contact persons in February 2021.

### 2.2. Survey

The questionnaire consists of nine questions that concern participant perception of self-competence regarding the care of patients with mental illness. The questionnaire was previously designed and used in a study of the educational program First Aid in Mental Illness [16], to measure perceived competence. The response options are fixed and given on a five-point scale from ‘not at all’ (1) to ‘a very large extent’ (5) (Appendix A).

The questionnaire was tested before distribution by three experienced ambulance nurses who otherwise did not participate in the study. These nurses judged the questionnaire as having good readability and clarity, being easy to complete, and having relevance based on the study aim. No changes were needed after this testing step. In addition to the survey questions, demographic data were collected in the form of sex, level of academic education, years as a nurse, and years as a professional in ambulance care.

### 2.3. Data Analysis

The variables based on the questionnaire were classified as ordinal data and are presented in tables and figures as medians and interquartile ranges. Sex differences in years working as a nurse and in the ambulance service were analyzed using the parametric *t*-test and are presented as an arithmetic mean ± standard deviation. Proportional analyses were performed with non-parametric Chi^2^ tests regarding sex, level of education (RN vs. SN), and proportions of novices (<5 years in ambulance care) and experts (≥5 years in ambulance care) [13]. Relationship analyses (correlation coefficients, *r*) were performed between questionnaire responses and the level of education and classification as novice or expert. These bi-variable correlation analyses were performed with Spearman’s rank correlation.

Finally, a question-index value (Q-IV; range: 0–1.0) was defined as a summary of the proportion of positive responses (%), defined as a rating from ‘to a quite large extent’ to ‘to a very large extent’ (Appendix A). This value was analyzed by sex and overall self-perception. Ranges of these index values were taken to define self-perceived competence as follows (inspired by Altman [17]): <0.20, none or very bad; 0.21–0.40, bad; 0.41–0.60, fairly good; 0.61–0.80, good; and 0.81–1.00, very good. Collected data were analyzed using SPSS^®^ (Statistical Package for the Social Sciences) version 26.0.

## 3. Results

The results are based on a response rate of 40% (*n* = 124) of distributed questionnaires (total, *n* = 311; Table 1). Response rates were similar between men and women (51% vs. 49%, *p* = 0.923). Most of the informants were SNs (86%, *p* = 0.001), and men reported significantly longer experience in ambulance care compared with women (9 ± 7 vs. 6 ± 4 years, *p* = 0.020). Most respondents (90%) were experts (*p* = 0.006).

As the data in Table 2 suggest, there were non-significant linear relationships between competence rankings and the level of education (RN vs. SN: *r* = −0.036–0.128, *p* = 0.236–0.738) and professional experience (novice vs. expert, *r* = 0.028 to −0.110, *p* = 0.236–0.773).

**Figure 1:**
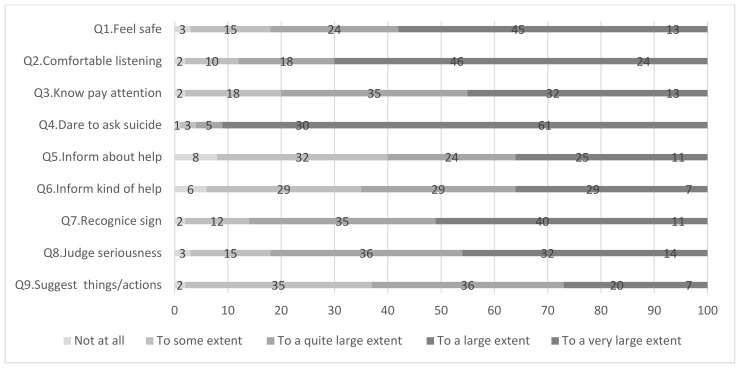
Questions and relative response rates based on a five-point scale, ranging from 1 = ‘not at all’ to 5 = ‘to a very large extent’.

As Figure 1 indicates, a clear majority of the respondents ask patients with mental illness if they have suicidal thoughts (Q4: 61%, ‘to a large extent’). On the other hand, response rates for being able to provide information (Q5) and propose further measures (Q9) showed that the informants rated their abilities lower (‘not at all’ and ‘to some extent’, 40% and 37%).

**Figure 2:**
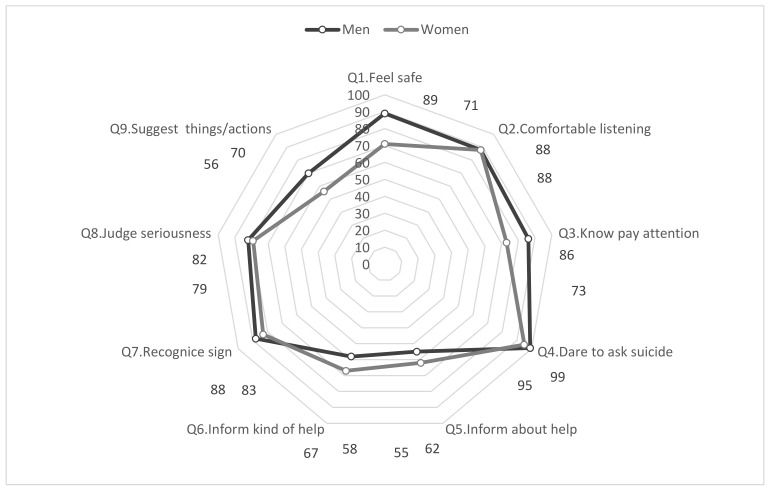
Response differences between men and women regarding the valuation of relative frequencies of positive response rates (Q-IV, response rates estimated according to a range from ‘to a fairly large extent’ through ‘to a very large extent’).

In Figure 2, the Q-IV values indicate that on six of the nine questions (questions 1, 3, 4, 7, 8, and 9), women rated their abilities slightly lower than men. The second highest estimate of abilities was for Q2, reporting on feeling comfortable listening and talking (men 88% vs. women 88%). The lowest positive response rate was for men, regarding Q5, to be able to provide information and help (55%).

As Table 3 shows, the informants had an overall good perception (mean Q-IV, 0.80) of their competence in the care of patients with mental illness.

## 4. Discussion

The purpose of this study was to evaluate how ambulance nurses perceive their competence in the care of patients with mental illness. Our results demonstrate an overall good perception, although the respondents perceived shortcomings in their knowledge in certain areas. Women rated their knowledge and skills slightly lower than did men, and higher education level or lengthier professional experience did not seem to affect self-perceived competence. Previous studies have explored how ambulance staff view their education in managing mental illness [18,19], indicating that paramedics often felt inadequately prepared in cases related to mental health [5,20]. Our results point to a similar phenomenon in education and training curricula for ambulance nurses in Sweden.

Ambulance care often is a patient’s first contact in the care chain, so it is important for their safety that they be assessed and treated correctly. The situation can be demanding, and special skills are required to meet the complex situations involved in caring for a patient with mental illness. Our findings suggest that respondents felt shortcomings in certain areas of knowledge, but we interpret this result largely to involve determining where certain patients should be triaged. Sweden has many levels of open clinics, together with traditional psychiatric clinics, which probably complicates triage decision-making for ambulance staff [21]. Another obvious concern is that non-conveyed patients represent a significant proportion of all patients cared for by ambulance services. Lederman et al. concluded that, in general, most cases of non-conveyance occur at the highest dispatch level, largely involve younger patients, and often are related to psychiatric symptoms [22]. Increased theoretical knowledge, as well as access to previous medical records, would probably lead to an increased sense of security if certain patients are judged as non-conveyed.

Todorova et al. examined the level of knowledge among ambulance nurses before they began a prehospital acute psychiatry project [7]. Their study revealed that ambulance nurses urgently needed extended knowledge on psychiatric mental disorders to recognize and provide the appropriate level of care and referral to appropriate departments. These participants perceived that the reason for the gap was the focus of the specialist nursing curriculum on managing medical illnesses, so that mental symptoms were often overlooked. Most participants in our study were SNs, and our correlation results showed no associations, suggesting that education level was not a significant factor in self-perception of competence in the care of patients with mental illness.

Overall, our study further describes a marginal sex difference in overall emergency preparedness for handling issues related to mental health. These findings are in line with those of Emund et al., who found that among paramedics in Australia, women were less confident than their male counterparts in handling mental illness, especially those related to anxious behaviors [23]. Mason cited female leadership and a culture of gender in ambulance care as an explanation for these differences [24], which we think could also apply in Sweden. Another factor may be that the women who responded to the questionnaire have felt less comfortable in these situations because ambulance staff had often been threatened by a patient or close relative [10].

Perrone McIntosh also found that nurses in emergency care did not know what skills were expected of them in the care of patients with mental illnesses. It can therefore be assumed that estimating one’s competence can be difficult without knowledge of what is expected [9]. Jansson et al. showed that practitioners with less education in ambulance care rated their competence higher than those with a higher education level [3]. The same phenomenon may be present in our study, but Daggenvoorde et al. reported that education level does not always improve professional skills [25]. Professional competence is the ability to use knowledge and skills in a certain professional context [10], but environment, personal abilities, self-confidence, professional opportunities, and reflection can affect the transfer of theoretical knowledge and clinical skills to useful professional competence. The present study does not examine the ambulance nurses’ attitudes regarding mental illness, but in both Swedish and foreign contexts it has been described that biomedical conditions and diagnosis are more natural to work with. One reason that can be mentioned may be the stigma that exists in society, of which nurses are a part, around mental illness [18,26,27]. Thoroughly defining a professional competence is thus complex because it can be seen as consisting of several separate specific knowledge areas required within a professional specialty [28].

A curriculum for a specialist nursing program should reflect the actual knowledge, competence, and skills required in the profession-specific environment and be based on a thorough description [29]. Today, the differentiated curriculum is perceived to be focused on the medical knowledge required to treat and care for patients in specific environments [30]. Shortcomings in educational content in the SN curriculum may be among the reasons for lower self-rated competence in caring for patients with mental illnesses. Judging by our results, it is reasonable to assume that educational efforts in ambulance care need to be intensified to provide nurses with broader knowledge about mental illness and the kind of assistance healthcare can offer these patients. We have previously cited that professional competence often is influenced by the professional attitudes, approaches, and perceptions found within the profession itself [14]. Simulated training together with the psychiatric health departments can, therefore, be beneficial for nurses in ambulance care. In connection with extended educational initiatives, an effect of sex should also be considered to increase security for female nurses. Further studies are needed to evaluate whether extended educational efforts can increase the experience of security and satisfaction in this context.

### Limitations

Questionnaire surveys normally yield a low response rate, and studies in Sweden indicate that responses will be received from up to 75% of people over the age of 70, compared to only 40% of persons of 20–24 years of age [31]. However, we believe that low response rates are not necessarily a major cause for concern because a more important factor is that the respondents are representative of the target group being investigated. Our results are likely an authentic reflection of this population, in part because the strengths of this study include a random representation of a regional sample of ambulance nurses who completed the survey confidentially. Nevertheless, the inability to capture responses from all targeted informants may have produced a misleading picture of the context.

Another limitation that it is necessary to reflect is the meaning of self-perceived competence. As we do not know how the informants separate subjective competence from objectively perceived competence, our results must be interpreted with a certain caution. Still, our main findings are in line with the referenced results in other countries.

## 5. Conclusions

Our findings suggest that ambulance nurses perceive their overall competencies in the encounter with patients with mental illness as good. However, the nurses perceived shortcomings in certain knowledge areas, and the results identified slight sex differences. Higher academic education level or lengthier professional experience does not seem to affect perceived confidence and preparedness in caring for patients with mental illnesses. Therefore, extended collaboration with central psychiatric departments, access to previous records, and workforce requirements aligned to the different nurse curricula are probably required to support triage of patients to the right level of care.

## Figures and Tables

**Table 1:** Absolute and relative frequencies by sex, education (registered nurse vs. specialist nurse), years as a nurse, years in ambulance service, and novice (<5 years) vs. expert (≥5 years).

Respondent	*n* = 124	*p*
Sex (male/female), *n* (%)	55 (51)/53 (49)	0.923 ^a^
Missing values, *n* (%)	16 (13)	
Education (registered/specialist nurse), *n* (%)	17 (14)/71 (86)	0.001 ^a,^*
Missing values, *n* (%)	36 (29)	
Years as nurse (men/women), mean ± SD	13 ± 7/13 ± 7	0.919 ^b^
Years as nurse (men/women), min–max	1–37/2–37	
Years in ambulance service (men/women), mean ± SD	9 ± 7/6 ± 4	0.020 ^b,^*
Years in ambulance service (men/women), min–max	1–32/1–17	
Novice vs. expert, *n* (%)	12 (10)/107 (90)	0.006 ^a,^*

^a^ Non-parametric Chi^2^ test; ^b^ *t*-test; * alpha value, *p* < 0.05.

**Table 2:** Response rates for each question and correlation coefficients (*r*)/*p* values for the association of self-perceived competence rankings with education (registered vs. specialist nurses) and with professional experience (novice, <5 years vs. expert, ≥5 years) in the ambulance service.

Question	Response Rates Median (Interquartile Range)	Response Rates Min–max	Registered/Specialist Nurse *r/p*	Novice/Expert *r/p*
Q1. Feel safe	4 (3–4)	1–5	−0.036/0.738	0.065/0.481
Q2. Comfortable listening	4 (3–4)	1–5	−0.128/0.236	0.028/0.765
Q3. Know to pay attention	3 (3–4)	1–5	−0.066/0.541	−0.059/0.526
Q4. Dare to ask about suicide	5 (4–5)	1–5	0.104/0.339	−0.045/0.632
Q5. Inform about help	3 (2–4)	1–5	−0.090/0.407	−0.110/0.236
Q6. Inform about kind of help	3 (2–4)	1–5	−0.108/0.316	−0.062/0.506
Q7. Recognize signs	3.5 (3–4)	1–5	−0.036/0.739	−0.080/0.385
Q8. Judge seriousness	3 (3–4)	1–5	−0.048/0.654	−0.027/0.773
Q9. Suggest things/actions	3 (2–4)	1–5	−0.081/0.453	−0.073/0.433

**Table 3:** Question index values (Q-IV), indicating self-perceived competence *.

Question	Q-IV	Self-Perception of Competence
Q1. I feel safe with a person who does not seem to feel mentally good.	0.89, men 0.71, women	Very good Good
Q2. I am comfortable listening and talking to a person about their mental health problems.	0.88, men 0.88, women	Very good Very good
Q3. I know what to listen to and pay attention to when I talk to someone who is down and sad.	0.86, men 0.73, women	Very good Good
Q4. I dare to ask if anyone has thoughts of taking their life/committing suicide.	0.99, men 0.95, women	Very good Very good
Q5. I can give people who are mentally ill information about what effective help is available.	0.62, men 0.55, women	Good Fairly good
Q6. I can inform them where to turn for the right kind of help.	0.58, men 0.67, women	Fairly good Good
Q7. I recognize signs that a person is suffering from a mental illness.	0.88, men 0.83, women	Very good Very good
Q8. I can judge the seriousness of a situation where a person is in a severe mental crisis.	0.82, men 0.79, women	Very good Good
Q9. I can suggest things/actions that can make a person with mental illness feel better.	0.70, men 0.56, women	Good Fairly good
Overall perception (mean Q-IV)	**0.80, overall** 0.89, men 0.71, women	**Good** Very good Good

* Ranges for perceived competence: <0.20, no or very bad; 0.21–0.40, bad; 0.41–0.60, fairly good; 0.61–0.80, good; and 0.81–1.00, very good.

## Data Availability

The datasets generated and analyzed during this study are not publicly available to protect the personal integrity of the nurses, but are available in redacted form from the corresponding author on reasonable request.

## Supplementary Materials

The following are available online at https://www.mdpi.com/article/10.3390/nursrep12010023/s1, File S1: STROBE Statement—Checklist of items that should be included in reports of cross-sectional studies.

## Appendix A

**Questionnaire survey**

**Answer options**

Not at all; To some extent; To a quite large extent; To a large extent; and To a very large extent

**Questions 1–9**

Q1. I feel safe with a person who does not seem to feel good mentally.

Q2. I am comfortable listening and talking to a person about their mental health problems.

Q3. I know what to listen to and pay attention to when I talk to someone who is down and sad.

Q4. I dare to ask if a person has thoughts of taking their own life/committing suicide.

Q5. I can give people who are mentally ill information about what types of effective help are available.

Q6. I can inform a person where to turn for the right kind of help.

Q7. I recognize signs that a person has a mental illness.

Q8. I can judge the seriousness of a situation when a person is in a severe mental crisis.

Q9. I can suggest things/actions that can comfort a person with a mental illness.
